# Diaqua­bis­[5-(2-pyridyl­meth­yl)tetra­zol­ato-κ^2^ *N* ^1^,*N* ^5^]zinc(II)

**DOI:** 10.1107/S1600536811019507

**Published:** 2011-05-28

**Authors:** Yang Liu, Ya-Ling Li, Xiu-Guang Wang, En-Cui Yang

**Affiliations:** a College of Chemistry, Tianjin Key Laboratory of Structure and Performance for Functional Molecules, Tianjin Normal University, Tianjin 300387, People’s Republic of China

## Abstract

In the title mononuclear complex, [Zn(C_7_H_6_N_5_)_2_(H_2_O)_2_], the Zn^II^ atom, located on an inversion centre, is in a distorted octa­hedral coordination geometry formed by four N atoms from two chelating 5-(2-pyridyl­meth­yl)tetra­zolate ligands and two O donors from two water mol­ecules. Inter­molecular O—H⋯N hydrogen bonds between the coordinated water mol­ecule and the tetra­zolyl group of the 5-(2-pyridyl­meth­yl)tetra­zolate ligand lead to the formation of a three-dimensional network.

## Related literature

For metal-organic frameworks with tetra­zolate ligands and their applications in magnetism, fluorescence and gas storage, see: Yang *et al.* (2011 ▶); Feng *et al.* (2010 ▶); Zhao *et al.* (2008 ▶); Panda *et al.* (2011 ▶). For metal complexes with *in situ-*generated 5-(2-pyridyl­meth­yl)-tetra­zolate ligands, see: Xu *et al.* (2009 ▶); Wang (2008 ▶).
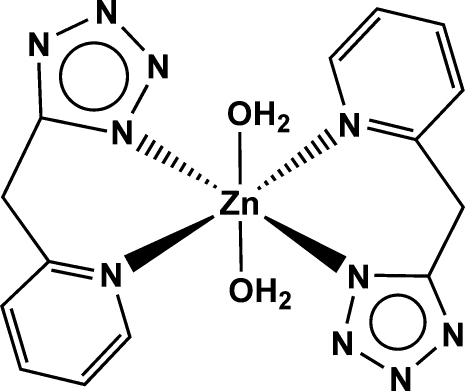


## Experimental

### 

#### Crystal data

[Zn(C_7_H_6_N_5_)_2_(H_2_O)_2_]
*M* *_r_* = 421.74
Monoclinic, 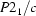

*a* = 6.6695 (4) Å
*b* = 13.8949 (8) Å
*c* = 10.8718 (5) Å
β = 127.055 (2)°
*V* = 804.05 (8) Å^3^
*Z* = 2
Mo *K*α radiation
μ = 1.57 mm^−1^
*T* = 173 K
0.20 × 0.10 × 0.08 mm

#### Data collection

Bruker APEXII CCD diffractometer
Absorption correction: multi-scan (*SADABS*; Sheldrick, 1996 ▶) *T*_min_ = 0.745, *T*_max_ = 0.885
3929 measured reflections
1388 independent reflections
1335 reflections with *I* > 2σ(*I*)
*R*_int_ = 0.030

#### Refinement

*R*[*F* ^2^ > 2σ(*F* ^2^)] = 0.022
*wR*(*F* ^2^) = 0.053
*S* = 1.05
1388 reflections
124 parameters
H-atom parameters constrained
Δρ_max_ = 0.62 e Å^−3^
Δρ_min_ = −0.30 e Å^−3^

### 

Data collection: *APEX2* (Bruker, 2003 ▶); cell refinement: *SAINT* (Bruker, 2001 ▶); data reduction: *SAINT*; program(s) used to solve structure: *SHELXS97* (Sheldrick, 2008 ▶); program(s) used to refine structure: *SHELXL97* (Sheldrick, 2008 ▶); molecular graphics: *SHELXTL* (Sheldrick, 2008 ▶) and *DIAMOND* (Brandenburg & Berndt, 1999 ▶); software used to prepare material for publication: *SHELXTL*.

## Supplementary Material

Crystal structure: contains datablocks I, global. DOI: 10.1107/S1600536811019507/bt5553sup1.cif

Structure factors: contains datablocks I. DOI: 10.1107/S1600536811019507/bt5553Isup2.hkl

Additional supplementary materials:  crystallographic information; 3D view; checkCIF report

## Figures and Tables

**Table 1:** Hydrogen-bond geometry (Å, °)

*D*—H⋯*A*	*D*—H	H⋯*A*	*D*⋯*A*	*D*—H⋯*A*
O1—H1*A*⋯N4^i^	0.85	2.00	2.8395 (19)	171
O1—H1*B*⋯N2^ii^	0.85	2.16	2.9386 (18)	152

Symmetry codes: (i) 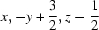; (ii) 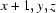.

**Fig. 1.**
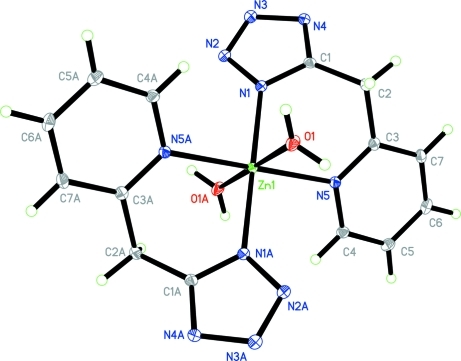
The molecular structure of the title complex with the atomic numbering scheme. Displacement ellipsoids were drawn at the 30% probability level [Symmetry code: (A) 1 – x, 2 – y, – z].

**Fig. 2.**
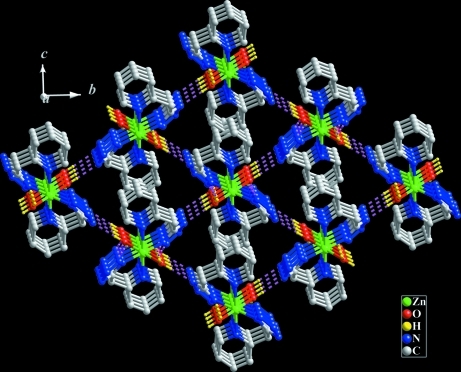
Three-dimensional network of the title complex assembled from hydrogen-bonding interactions.

## supplementary crystallographic information

### Comment

Design and construction of metal-organic frameworks (MOFs) with *in situ* generated tetrazolate ligands are of great interest due to their intriguing structures and topology (Zhao *et al.*, 2008), promising applications in magnetism (Yang *et al.* 2011), luminscence (Feng *et al.* 2010), and gas storage (Panda *et al.* 2011) as well as the effectiveness, simplicity, and environmental friendliness of the *in situ* synthetic route. Up to date, lots of tetrazolyl-based MOFs have been reported with special interest on the tuning of the organic nitrile and metal ions (Xu *et al.* 2009; Wang *et al.*, 2008). Herein, as our continuing investigations on the coordination chemistry of the tetrazolyl ligand, we report the crystal structure of a diaquazinc(II) complex with an *in situ* generated 5-(2-pyridylmethyl)-tetrazolate ligand.

The molecular structure of the title mononuclear complex is show Figure 1. The Zn^II^ ion in the mononuclear structure of I, locating on an inversion center, exhibits a slightly distorted octahedral geometry involoving four N donors from two in situ generated 5-(pyridin-2-ylmethyl)tetrazolate ligands, and two O atoms from a pair of coordinated water molecules. The flexible 5-(pyridin-2-ylmethyl)tetrazolate anion acts as a bidentate chelating ligand to coordinate with Zn^II^ through pyridyl and tetrazolyl N donors.

In the crystal structure,   intermolecular O—H···N hydrogen bonds between the coordinated water molecules and the tetrazolyl group of 5-(2-pyridylmethyl)- tetrazolate ligand (Table 2) lead to the formation of a three-dimensional network (Figure 2).

### Experimental

A mixture containing 2-(pyridin-2-yl)acetonitrile (26 mg, 0.2 mmol), Zn(NO_3_)_2_ (29.7 mg, 0.1 mmol), 1,3,5-benzenetricarboxylic acid (21.0 mg, 0.1 mmol), NaN_3_ (13.0 mg, 0.2 mmol), and doubly deionized water (10.0 ml) was sealed in a Teflon-lined reactor (23.0 ml) and heated at 125 °C for 72 h. After the mixture was cooled to room temperature at a rate of 5.5°C/h, pale-yellow block-shaped crystals suitable for X-ray diffraction analysis were obtained. Yield: 56% based on Zn^II^ salt. Anal. Calcd.for C_14_H_16_N_10_O_2_Zn: C, 39.87; H, 3.82; N, 33.21%. Found: C, 39.85; H, 3.82; N, 33.24%.

### Refinement

H atoms were located in a difference map but refined using a riding model with O-H = 0.85Å, C_aromatic_-H = 0.95Å, C_methylene_-H = 0.99Å and with U(H) set to 1.2 U of the parent atom.

### Crystal data

**Table:**

[Zn(C_7_H_6_N_5_)_2_(H_2_O)_2_]	*F*(000) = 432
*M**_r_* = 421.74	*D*_x_ = 1.742 Mg m^−^^3^
Monoclinic, *P*2_1_/*c*	Mo *K*α radiation, λ = 0.71073 Å
*a* = 6.6695 (4) Å	Cell parameters from 3869 reflections
*b* = 13.8949 (8) Å	θ = 2.8–28.4°
*c* = 10.8718 (5) Å	µ = 1.57 mm^−^^1^
β = 127.055 (2)°	*T* = 173 K
*V* = 804.05 (8) Å^3^	Block, pale yellow
*Z* = 2	0.20 × 0.10 × 0.08 mm

### Data collection

**Table:**

Bruker APEXII CCD diffractometer	1388 independent reflections
Radiation source: fine-focus sealed tube	1335 reflections with *I* > 2σ(*I*)
graphite	*R*_int_ = 0.030
φ and ω scans	θ_max_ = 25.0°, θ_min_ = 2.8°
Absorption correction: multi-scan (*SADABS*; Sheldrick, 1996)	*h* = −7→7
*T*_min_ = 0.745, *T*_max_ = 0.885	*k* = −16→11
3929 measured reflections	*l* = −12→12

### Refinement

**Table:**

Refinement on *F*^2^	Primary atom site location: structure-invariant direct methods
Least-squares matrix: full	Secondary atom site location: difference Fourier map
*R*[*F*^2^ > 2σ(*F*^2^)] = 0.022	Hydrogen site location: inferred from neighbouring sites
*wR*(*F*^2^) = 0.053	H-atom parameters constrained
*S* = 1.05	*w* = 1/[σ^2^(*F*_o_^2^) + (0.0146*P*)^2^ + 0.7405*P*] where *P* = (*F*_o_^2^ + 2*F*_c_^2^)/3
1388 reflections	(Δ/σ)_max_ < 0.001
124 parameters	Δρ_max_ = 0.62 e Å^−^^3^
0 restraints	Δρ_min_ = −0.30 e Å^−^^3^

### Special details

**Table:**

Geometry. All e.s.d.'s (except the e.s.d. in the dihedral angle between two l.s. planes) are estimated using the full covariance matrix. The cell e.s.d.'s are taken into account individually in the estimation of e.s.d.'s in distances, angles and torsion angles; correlations between e.s.d.'s in cell parameters are only used when they are defined by crystal symmetry. An approximate (isotropic) treatment of cell e.s.d.'s is used for estimating e.s.d.'s involving l.s. planes.
Refinement. Refinement of *F*^2^ against ALL reflections. The weighted *R*-factor *wR* and goodness of fit *S* are based on *F*^2^, conventional *R*-factors *R* are based on *F*, with *F* set to zero for negative *F*^2^. The threshold expression of *F*^2^ > σ(*F*^2^) is used only for calculating *R*-factors(gt) *etc*. and is not relevant to the choice of reflections for refinement. *R*-factors based on *F*^2^ are statistically about twice as large as those based on *F*, and *R*- factors based on ALL data will be even larger.

### Fractional atomic coordinates and isotropic or equivalent isotropic displacement parameters (Å^2^)

**Table:**

	*x*	*y*	*z*	*U*_iso_*/*U*_eq_	
Zn1	0.5000	1.0000	0.0000	0.01318 (11)	
O1	0.6481 (2)	0.90283 (9)	−0.08811 (14)	0.0196 (3)	
H1A	0.5809	0.8510	−0.1382	0.024*	
H1B	0.7995	0.9131	−0.0532	0.024*	
N1	0.4006 (3)	0.88555 (10)	0.07937 (16)	0.0144 (3)	
N2	0.1830 (3)	0.86762 (11)	0.05983 (17)	0.0164 (3)	
N3	0.2217 (3)	0.79864 (11)	0.15370 (17)	0.0185 (3)	
N4	0.4661 (3)	0.77039 (11)	0.23846 (16)	0.0161 (3)	
N5	0.8595 (3)	0.99858 (9)	0.22729 (17)	0.0135 (3)	
C1	0.5681 (3)	0.82608 (12)	0.18945 (19)	0.0140 (4)	
C2	0.8406 (3)	0.82446 (13)	0.2548 (2)	0.0166 (4)	
H2A	0.8553	0.8047	0.1730	0.020*	
H2B	0.9289	0.7759	0.3381	0.020*	
C3	0.9670 (3)	0.92064 (13)	0.3181 (2)	0.0146 (4)	
C4	0.9710 (3)	1.08445 (13)	0.2853 (2)	0.0159 (4)	
H4	0.8952	1.1398	0.2218	0.019*	
C5	1.1913 (3)	1.09628 (14)	0.4334 (2)	0.0189 (4)	
H5	1.2641	1.1582	0.4704	0.023*	
C6	1.3018 (3)	1.01590 (14)	0.5256 (2)	0.0196 (4)	
H6	1.4524	1.0216	0.6276	0.023*	
C7	1.1908 (3)	0.92742 (14)	0.4676 (2)	0.0175 (4)	
H7	1.2659	0.8712	0.5289	0.021*	

### Atomic displacement parameters (Å^2^)

**Table:**

	*U*^11^	*U*^22^	*U*^33^	*U*^12^	*U*^13^	*U*^23^
Zn1	0.01153 (16)	0.01131 (17)	0.01161 (16)	−0.00133 (10)	0.00427 (13)	0.00173 (10)
O1	0.0142 (6)	0.0174 (7)	0.0229 (6)	−0.0019 (5)	0.0089 (5)	−0.0076 (6)
N1	0.0128 (7)	0.0135 (7)	0.0140 (7)	−0.0012 (6)	0.0066 (6)	0.0001 (6)
N2	0.0138 (7)	0.0160 (8)	0.0174 (7)	−0.0018 (6)	0.0083 (6)	−0.0003 (7)
N3	0.0160 (7)	0.0183 (8)	0.0189 (7)	−0.0012 (6)	0.0093 (6)	0.0004 (7)
N4	0.0150 (7)	0.0145 (7)	0.0169 (7)	−0.0003 (6)	0.0086 (6)	0.0018 (6)
N5	0.0127 (7)	0.0136 (8)	0.0141 (7)	0.0005 (5)	0.0080 (6)	0.0011 (5)
C1	0.0160 (8)	0.0104 (8)	0.0143 (8)	−0.0005 (7)	0.0085 (7)	−0.0020 (7)
C2	0.0153 (8)	0.0139 (9)	0.0189 (9)	0.0028 (7)	0.0094 (7)	0.0029 (8)
C3	0.0141 (8)	0.0176 (9)	0.0164 (8)	0.0020 (7)	0.0115 (7)	0.0019 (8)
C4	0.0166 (8)	0.0159 (9)	0.0146 (8)	−0.0015 (7)	0.0092 (7)	0.0008 (8)
C5	0.0180 (9)	0.0212 (10)	0.0169 (9)	−0.0049 (8)	0.0102 (8)	−0.0038 (8)
C6	0.0127 (8)	0.0298 (10)	0.0151 (9)	0.0001 (8)	0.0078 (7)	−0.0005 (8)
C7	0.0145 (8)	0.0214 (10)	0.0172 (9)	0.0051 (7)	0.0100 (7)	0.0058 (8)

### Geometric parameters (Å, °)

**Table:**

Zn1—N1^i^	2.0983 (14)	N5—C3	1.345 (2)
Zn1—N1	2.0984 (14)	C1—C2	1.501 (2)
Zn1—N5	2.1714 (15)	C2—C3	1.507 (2)
Zn1—N5^i^	2.1714 (15)	C2—H2A	0.9900
Zn1—O1	2.2039 (12)	C2—H2B	0.9900
Zn1—O1^i^	2.2039 (12)	C3—C7	1.399 (2)
O1—H1A	0.8501	C4—C5	1.388 (2)
O1—H1B	0.8500	C4—H4	0.9500
N1—C1	1.324 (2)	C5—C6	1.380 (3)
N1—N2	1.3572 (19)	C5—H5	0.9500
N2—N3	1.307 (2)	C6—C7	1.376 (3)
N3—N4	1.360 (2)	C6—H6	0.9500
N4—C1	1.335 (2)	C7—H7	0.9500
N5—C4	1.345 (2)		
			
N1^i^—Zn1—N1	180.00 (7)	C3—N5—Zn1	125.26 (11)
N1^i^—Zn1—N5	93.96 (5)	N1—C1—N4	111.52 (15)
N1—Zn1—N5	86.04 (5)	N1—C1—C2	123.99 (15)
N1^i^—Zn1—N5^i^	86.04 (5)	N4—C1—C2	124.45 (15)
N1—Zn1—N5^i^	93.96 (5)	C1—C2—C3	112.78 (14)
N5—Zn1—N5^i^	180.0	C1—C2—H2A	109.0
N1^i^—Zn1—O1	87.13 (5)	C3—C2—H2A	109.0
N1—Zn1—O1	92.87 (5)	C1—C2—H2B	109.0
N5—Zn1—O1	90.71 (5)	C3—C2—H2B	109.0
N5^i^—Zn1—O1	89.29 (5)	H2A—C2—H2B	107.8
N1^i^—Zn1—O1^i^	92.87 (5)	N5—C3—C7	121.50 (16)
N1—Zn1—O1^i^	87.13 (5)	N5—C3—C2	118.36 (15)
N5—Zn1—O1^i^	89.29 (5)	C7—C3—C2	120.14 (16)
N5^i^—Zn1—O1^i^	90.71 (5)	N5—C4—C5	123.28 (17)
O1—Zn1—O1^i^	180.00 (6)	N5—C4—H4	118.4
Zn1—O1—H1A	126.6	C5—C4—H4	118.4
Zn1—O1—H1B	115.2	C6—C5—C4	118.38 (17)
H1A—O1—H1B	117.0	C6—C5—H5	120.8
C1—N1—N2	105.52 (13)	C4—C5—H5	120.8
C1—N1—Zn1	122.75 (11)	C7—C6—C5	119.06 (17)
N2—N1—Zn1	130.38 (11)	C7—C6—H6	120.5
N3—N2—N1	108.81 (13)	C5—C6—H6	120.5
N2—N3—N4	109.47 (13)	C6—C7—C3	119.69 (17)
C1—N4—N3	104.66 (14)	C6—C7—H7	120.2
C4—N5—C3	118.08 (15)	C3—C7—H7	120.2
C4—N5—Zn1	116.42 (11)		
			
N1^i^—Zn1—N1—C1	−100 (10)	O1^i^—Zn1—N5—C3	116.31 (13)
N5—Zn1—N1—C1	−26.79 (13)	N2—N1—C1—N4	1.07 (19)
N5^i^—Zn1—N1—C1	153.21 (13)	Zn1—N1—C1—N4	169.09 (11)
O1—Zn1—N1—C1	63.72 (13)	N2—N1—C1—C2	−176.72 (15)
O1^i^—Zn1—N1—C1	−116.28 (13)	Zn1—N1—C1—C2	−8.7 (2)
N1^i^—Zn1—N1—N2	65 (10)	N3—N4—C1—N1	−0.63 (19)
N5—Zn1—N1—N2	137.97 (14)	N3—N4—C1—C2	177.15 (15)
N5^i^—Zn1—N1—N2	−42.03 (14)	N1—C1—C2—C3	56.5 (2)
O1—Zn1—N1—N2	−131.52 (14)	N4—C1—C2—C3	−121.02 (18)
O1^i^—Zn1—N1—N2	48.48 (14)	C4—N5—C3—C7	−1.2 (2)
C1—N1—N2—N3	−1.10 (18)	Zn1—N5—C3—C7	−175.34 (12)
Zn1—N1—N2—N3	−167.85 (11)	C4—N5—C3—C2	178.95 (15)
N1—N2—N3—N4	0.75 (18)	Zn1—N5—C3—C2	4.8 (2)
N2—N3—N4—C1	−0.09 (18)	C1—C2—C3—N5	−51.8 (2)
N1^i^—Zn1—N5—C4	34.93 (13)	C1—C2—C3—C7	128.33 (16)
N1—Zn1—N5—C4	−145.07 (13)	C3—N5—C4—C5	0.3 (2)
N5^i^—Zn1—N5—C4	85.1 (7)	Zn1—N5—C4—C5	174.98 (13)
O1—Zn1—N5—C4	122.11 (12)	N5—C4—C5—C6	0.2 (3)
O1^i^—Zn1—N5—C4	−57.89 (12)	C4—C5—C6—C7	0.2 (3)
N1^i^—Zn1—N5—C3	−150.86 (13)	C5—C6—C7—C3	−1.0 (3)
N1—Zn1—N5—C3	29.14 (13)	N5—C3—C7—C6	1.6 (2)
N5^i^—Zn1—N5—C3	−100.7 (7)	C2—C3—C7—C6	−178.58 (16)
O1—Zn1—N5—C3	−63.69 (13)		

Symmetry codes: (i) −*x*+1, −*y*+2, −*z*.

### Hydrogen-bond geometry (Å, °)

**Table:**

*D*—H···*A*	*D*—H	H···*A*	*D*···*A*	*D*—H···*A*
O1—H1A···N4^ii^	0.85	2.00	2.8395 (19)	171
O1—H1B···N2^iii^	0.85	2.16	2.9386 (18)	152

Symmetry codes: (ii) *x*, −*y*+3/2, *z*−1/2; (iii) *x*+1, *y*, *z*.
